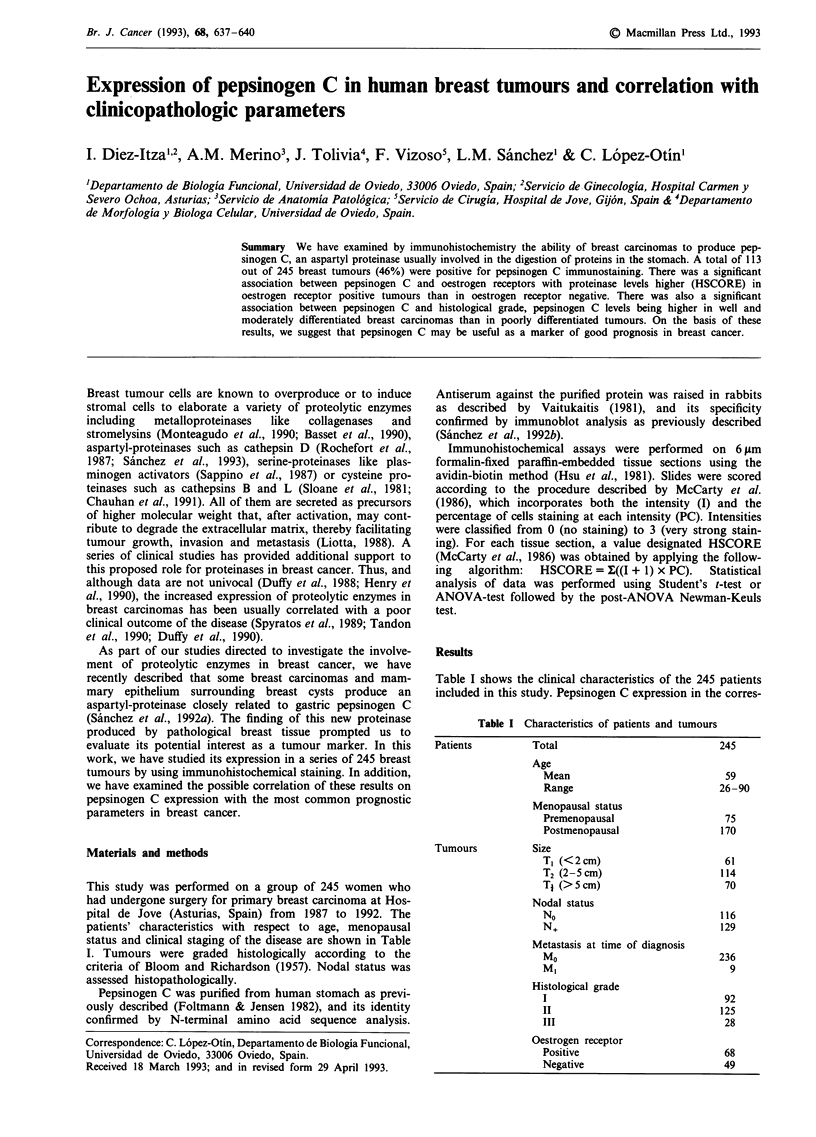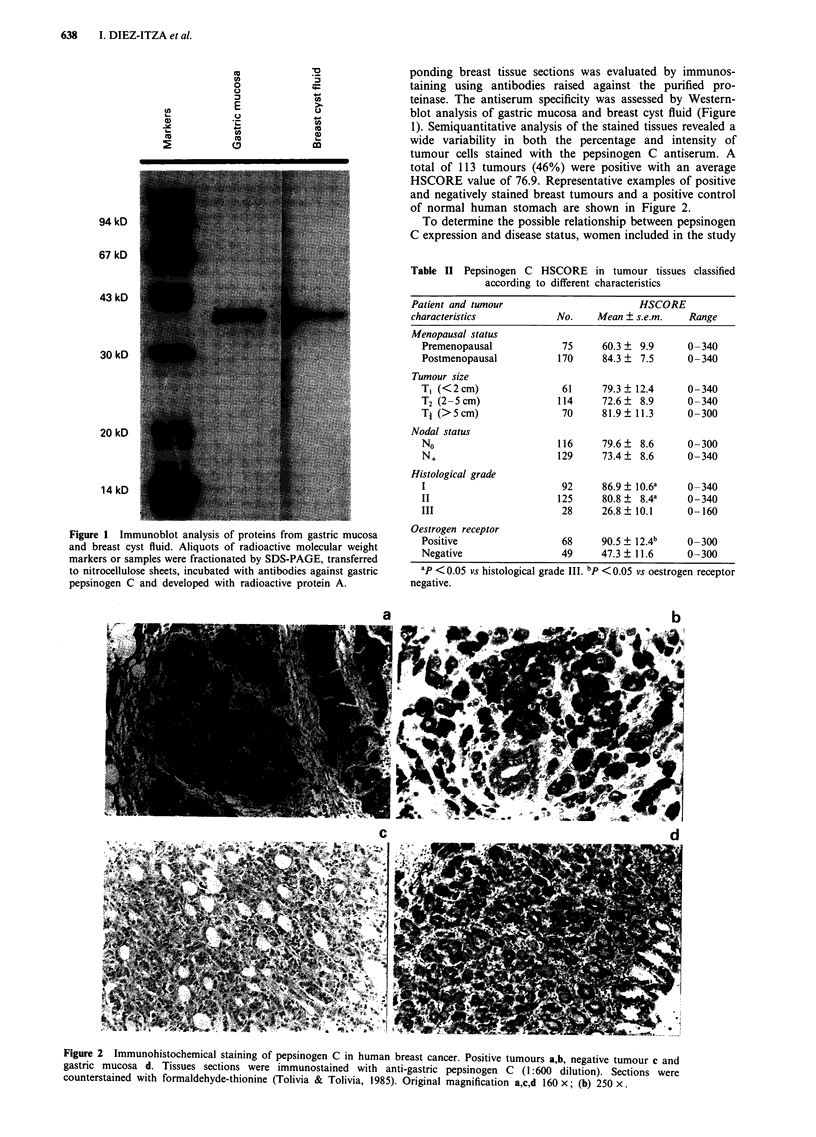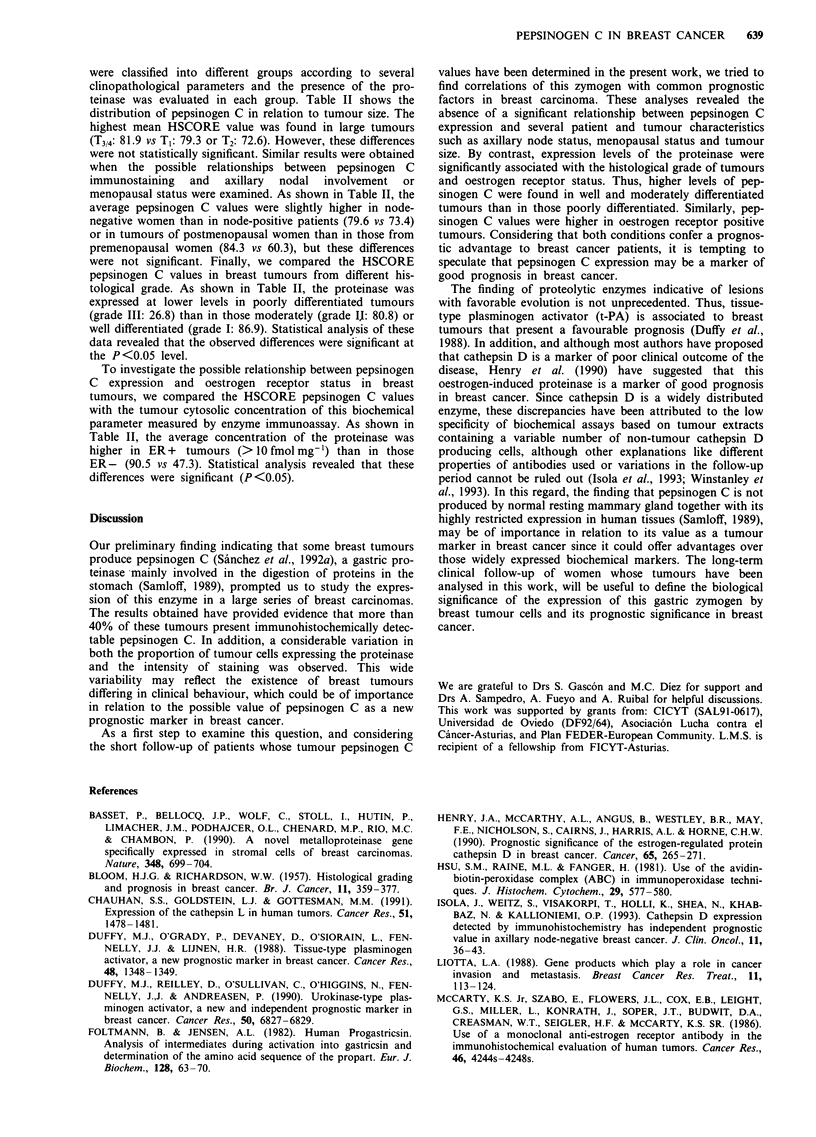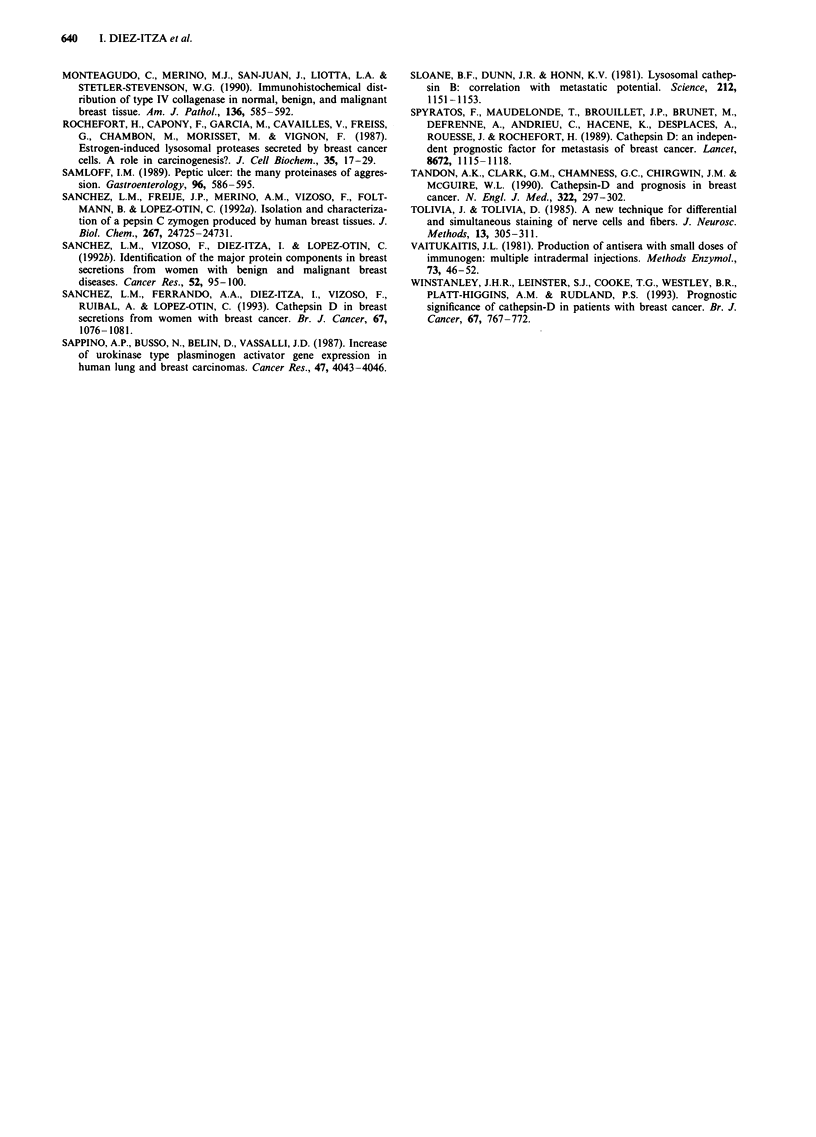# Expression of pepsinogen C in human breast tumours and correlation with clinicopathologic parameters.

**DOI:** 10.1038/bjc.1993.399

**Published:** 1993-09

**Authors:** I. Diez-Itza, A. M. Merino, J. Tolivia, F. Vizoso, L. M. Sánchez, C. López-Otín

**Affiliations:** Departamento de Biología Funcional, Universidad de Oviedo, Spain.

## Abstract

**Images:**


Br. J. Cancer (1993), 68, 637-640 © Macmillan Press Ltd., 1993

### Expression of pepsinogen C in human breast tumours and correlation with clinicopathologic parameters

I. Diez-Itza[1,2], A.M. Merino[3], J. Tolivia[4], F. Vizoso[5], L.M. Sánchez[1] & C. López-Otín[1]

[1]*Departamento de Biología Funcional, Universidad de Oviedo, 33006 Oviedo, Spain;* [2]*Servicio de Ginecología, Hospital Carmen y Severo Ochoa, Asturias;* [3]*Servicio de Anatomía Patológica;* [5]*Servicio de Cirugía, Hospital de Jove, Gijón, Spain &* [4]*Departamento de Morfología y Biologa Celular, Universidad de Oviedo, Spain.*

**Summary** We have examined by immunohistochemistry the ability of breast carcinomas to produce pepsinogen C, an aspartyl proteinase usually involved in the digestion of proteins in the stomach. A total of 113 out of 245 breast tumours (46%) were positive for pepsinogen C immunostaining. There was a significant association between pepsinogen C and oestrogen receptors with proteinase levels higher (HSCORE) in oestrogen receptor positive tumours than in oestrogen receptor negative. There was also a significant association between pepsinogen C and histological grade, pepsinogen C levels being higher in well and moderately differentiated breast carcinomas than in poorly differentiated tumours. On the basis of these results, we suggest that pepsinogen C may be useful as a marker of good prognosis in breast cancer.

Breast tumour cells are known to overproduce or to induce stromal cells to elaborate a variety of proteolytic enzymes including metalloproteinases like collagenases and stromelysins (Monteagudo *et al.*, 1990; Basset *et al.*, 1990), aspartyl-proteinases such as cathepsin D (Rochefort *et al.*, 1987; Sánchez *et al.*, 1993), serine-proteinases like plasminogen activators (Sappino *et al.*, 1987) or cysteine proteinases such as cathepsins B and L (Sloane *et al.*, 1981; Chauhan *et al.*, 1991). All of them are secreted as precursors of higher molecular weight that, after activation, may contribute to degrade the extracellular matrix, thereby facilitating tumour growth, invasion and metastasis (Liotta, 1988). A series of clinical studies has provided additional support to this proposed role for proteinases in breast cancer. Thus, and although data are not univocal (Duffy *et al.*, 1988; Henry *et al.*, 1990), the increased expression of proteolytic enzymes in breast carcinomas has been usually correlated with a poor clinical outcome of the disease (Spyratos *et al.*, 1989; Tandon *et al.*, 1990; Duffy *et al.*, 1990).

As part of our studies directed to investigate the involvement of proteolytic enzymes in breast cancer, we have recently described that some breast carcinomas and mammary epithelium surrounding breast cysts produce an aspartyl-proteinase closely related to gastric pepsinogen C (Sánchez *et al.*, 1992*a*). The finding of this new proteinase produced by pathological breast tissue prompted us to evaluate its potential interest as a tumour marker. In this work, we have studied its expression in a series of 245 breast tumours by using immunohistochemical staining. In addition, we have examined the possible correlation of these results on pepsinogen C expression with the most common prognostic parameters in breast cancer.

### Materials and methods

This study was performed on a group of 245 women who had undergone surgery for primary breast carcinoma at Hospital de Jove (Asturias, Spain) from 1987 to 1992. The patients' characteristics with respect to age, menopausal status and clinical staging of the disease are shown in Table I. Tumours were graded histologically according to the criteria of Bloom and Richardson (1957). Nodal status was assessed histopathologically.

Pepsinogen C was purified from human stomach as previously described (Foltmann & Jensen 1982), and its identity confirmed by N-terminal amino acid sequence analysis. Antiserum against the purified protein was raised in rabbits as described by Vaitukaitis (1981), and its specificity confirmed by immunoblot analysis as previously described (Sánchez *et al.*, 1992*b*).

Immunohistochemical assays were performed on 6 μm formalin-fixed paraffin-embedded tissue sections using the avidin-biotin method (Hsu *et al.*, 1981). Slides were scored according to the procedure described by McCarty *et al.* (1986), which incorporates both the intensity (I) and the percentage of cells staining at each intensity (PC). Intensities were classified from 0 (no staining) to 3 (very strong staining). For each tissue section, a value designated HSCORE (McCarty *et al.*, 1986) was obtained by applying the following algorithm: $HSCORE = \Sigma((I + 1) \times PC)$. Statistical analysis of data was performed using Student's *t*-test or ANOVA-test followed by the post-ANOVA Newman-Keuls test.

### Results

Table I shows the clinical characteristics of the 245 patients included in this study. Pepsinogen C expression in the corres-

**Table I** Characteristics of patients and tumours

| | | |
|---|---|---|
| Patients | Total | 245 |
| | Age | |
| | Mean | 59 |
| | Range | 26-90 |
| | Menopausal status | |
| | Premenopausal | 75 |
| | Postmenopausal | 170 |
| Tumours | Size | |
| | $T_1$ (<2 cm) | 61 |
| | $T_2$ (2-5 cm) | 114 |
| | $T_3$ (>5 cm) | 70 |
| | Nodal status | |
| | $N_0$ | 116 |
| | $N_+$ | 129 |
| | Metastasis at time of diagnosis | |
| | $M_0$ | 236 |
| | $M_1$ | 9 |
| | Histological grade | |
| | I | 92 |
| | II | 125 |
| | III | 28 |
| | Oestrogen receptor | |
| | Positive | 68 |
| | Negative | 49 |

Correspondence: C. López-Otín, Departamento de Biología Funcional, Universidad de Oviedo, 33006 Oviedo, Spain.
Received 18 March 1993; and in revised form 29 April 1993.

**Figure 1** Immunoblot analysis of proteins from gastric mucosa and breast cyst fluid. Aliquots of radioactive molecular weight markers or samples were fractionated by SDS-PAGE, transferred to nitrocellulose sheets, incubated with antibodies against gastric pepsinogen C and developed with radioactive protein A.

ponding breast tissue sections was evaluated by immunostaining using antibodies raised against the purified proteinase. The antiserum specificity was assessed by Western-blot analysis of gastric mucosa and breast cyst fluid (Figure 1). Semiquantitative analysis of the stained tissues revealed a wide variability in both the percentage and intensity of tumour cells stained with the pepsinogen C antiserum. A total of 113 tumours (46%) were positive with an average HSCORE value of 76.9. Representative examples of positive and negatively stained breast tumours and a positive control of normal human stomach are shown in Figure 2.

To determine the possible relationship between pepsinogen C expression and disease status, women included in the study

**Table II** Pepsinogen C HSCORE in tumour tissues classified according to different characteristics

| *Patient and tumour characteristics* | *No.* | *HSCORE* Mean ± s.e.m. | *Range* |
|---|---|---|---|
| *Menopausal status* | | | |
| Premenopausal | 75 | 60.3 ± 9.9 | 0–340 |
| Postmenopausal | 170 | 84.3 ± 7.5 | 0–340 |
| *Tumour size* | | | |
| $T_1$ (<2 cm) | 61 | 79.3 ± 12.4 | 0–340 |
| $T_2$ (2–5 cm) | 114 | 72.6 ± 8.9 | 0–340 |
| $T_3$ (>5 cm) | 70 | 81.9 ± 11.3 | 0–300 |
| *Nodal status* | | | |
| $N_0$ | 116 | 79.6 ± 8.6 | 0–300 |
| $N_+$ | 129 | 73.4 ± 8.6 | 0–340 |
| *Histological grade* | | | |
| I | 92 | 86.9 ± 10.6[a] | 0–340 |
| II | 125 | 80.8 ± 8.4[a] | 0–340 |
| III | 28 | 26.8 ± 10.1 | 0–160 |
| *Oestrogen receptor* | | | |
| Positive | 68 | 90.5 ± 12.4[b] | 0–300 |
| Negative | 49 | 47.3 ± 11.6 | 0–300 |

[a]$P < 0.05$ *vs* histological grade III. [b]$P < 0.05$ *vs* oestrogen receptor negative.

**Figure 2** Immunohistochemical staining of pepsinogen C in human breast cancer. Positive tumours **a,b**, negative tumour **c** and gastric mucosa **d**. Tissues sections were immunostained with anti-gastric pepsinogen C (1:600 dilution). Sections were counterstained with formaldehyde-thionine (Tolivia & Tolivia, 1985). Original magnification **a,c,d** 160 ×; (**b**) 250 ×.

were classified into different groups according to several clinopathological parameters and the presence of the proteinase was evaluated in each group. Table II shows the distribution of pepsinogen C in relation to tumour size. The highest mean HSCORE value was found in large tumours ($T_{3/4}$: 81.9 *vs* $T_1$: 79.3 or $T_2$: 72.6). However, these differences were not statistically significant. Similar results were obtained when the possible relationships between pepsinogen C immunostaining and axillary nodal involvement or menopausal status were examined. As shown in Table II, the average pepsinogen C values were slightly higher in node-negative women than in node-positive patients (79.6 *vs* 73.4) or in tumours of postmenopausal women than in those from premenopausal women (84.3 *vs* 60.3), but these differences were not significant. Finally, we compared the HSCORE pepsinogen C values in breast tumours from different histological grade. As shown in Table II, the proteinase was expressed at lower levels in poorly differentiated tumours (grade III: 26.8) than in those moderately (grade II: 80.8) or well differentiated (grade I: 86.9). Statistical analysis of these data revealed that the observed differences were significant at the $P<0.05$ level.

To investigate the possible relationship between pepsinogen C expression and oestrogen receptor status in breast tumours, we compared the HSCORE pepsinogen C values with the tumour cytosolic concentration of this biochemical parameter measured by enzyme immunoassay. As shown in Table II, the average concentration of the proteinase was higher in ER+ tumours ($>10\,\mathrm{fmol\,mg^{-1}}$) than in those ER− (90.5 *vs* 47.3). Statistical analysis revealed that these differences were significant ($P<0.05$).

### Discussion

Our preliminary finding indicating that some breast tumours produce pepsinogen C (Sánchez *et al.*, 1992*a*), a gastric proteinase mainly involved in the digestion of proteins in the stomach (Samloff, 1989), prompted us to study the expression of this enzyme in a large series of breast carcinomas. The results obtained have provided evidence that more than 40% of these tumours present immunohistochemically detectable pepsinogen C. In addition, a considerable variation in both the proportion of tumour cells expressing the proteinase and the intensity of staining was observed. This wide variability may reflect the existence of breast tumours differing in clinical behaviour, which could be of importance in relation to the possible value of pepsinogen C as a new prognostic marker in breast cancer.

As a first step to examine this question, and considering the short follow-up of patients whose tumour pepsinogen C values have been determined in the present work, we tried to find correlations of this zymogen with common prognostic factors in breast carcinoma. These analyses revealed the absence of a significant relationship between pepsinogen C expression and several patient and tumour characteristics such as axillary node status, menopausal status and tumour size. By contrast, expression levels of the proteinase were significantly associated with the histological grade of tumours and oestrogen receptor status. Thus, higher levels of pepsinogen C were found in well and moderately differentiated tumours than in those poorly differentiated. Similarly, pepsinogen C values were higher in oestrogen receptor positive tumours. Considering that both conditions confer a prognostic advantage to breast cancer patients, it is tempting to speculate that pepsinogen C expression may be a marker of good prognosis in breast cancer.

The finding of proteolytic enzymes indicative of lesions with favorable evolution is not unprecedented. Thus, tissue-type plasminogen activator (t-PA) is associated to breast tumours that present a favourable prognosis (Duffy *et al.*, 1988). In addition, and although most authors have proposed that cathepsin D is a marker of poor clinical outcome of the disease, Henry *et al.* (1990) have suggested that this oestrogen-induced proteinase is a marker of good prognosis in breast cancer. Since cathepsin D is a widely distributed enzyme, these discrepancies have been attributed to the low specificity of biochemical assays based on tumour extracts containing a variable number of non-tumour cathepsin D producing cells, although other explanations like different properties of antibodies used or variations in the follow-up period cannot be ruled out (Isola *et al.*, 1993; Winstanley *et al.*, 1993). In this regard, the finding that pepsinogen C is not produced by normal resting mammary gland together with its highly restricted expression in human tissues (Samloff, 1989), may be of importance in relation to its value as a tumour marker in breast cancer since it could offer advantages over those widely expressed biochemical markers. The long-term clinical follow-up of women whose tumours have been analysed in this work, will be useful to define the biological significance of the expression of this gastric zymogen by breast tumour cells and its prognostic significance in breast cancer.

We are grateful to Drs S. Gascón and M.C. Díez for support and Drs A. Sampedro, A. Fueyo and A. Ruibal for helpful discussions. This work was supported by grants from: CICYT (SAL91-0617), Universidad de Oviedo (DF92/64), Asociación Lucha contra el Cáncer-Asturias, and Plan FEDER-European Community. L.M.S. is recipient of a fellowship from FICYT-Asturias.

### References

BASSET, P., BELLOCQ, J.P., WOLF, C., STOLL, I., HUTIN, P., LIMACHER, J.M., PODHAJCER, O.L., CHENARD, M.P., RIO, M.C. & CHAMBON, P. (1990). A novel metalloproteinase gene specifically expressed in stromal cells of breast carcinomas. *Nature*, **348**, 699–704.

BLOOM, H.J.G. & RICHARDSON, W.W. (1957). Histological grading and prognosis in breast cancer. *Br. J. Cancer*, **11**, 359–377.

CHAUHAN, S.S., GOLDSTEIN, L.J. & GOTTESMAN, M.M. (1991). Expression of the cathepsin L in human tumors. *Cancer Res.*, **51**, 1478–1481.

DUFFY, M.J., O'GRADY, P., DEVANEY, D., O'SIORAIN, L., FENNELLY, J.J. & LIJNEN, H.R. (1988). Tissue-type plasminogen activator, a new prognostic marker in breast cancer. *Cancer Res.*, **48**, 1348–1349.

DUFFY, M.J., REILLEY, D., O'SULLIVAN, C., O'HIGGINS, N., FENNELLY, J.J. & ANDREASEN, P. (1990). Urokinase-type plasminogen activator, a new and independent prognostic marker in breast cancer. *Cancer Res.*, **50**, 6827–6829.

FOLTMANN, B. & JENSEN, A.L. (1982). Human Progastricsin. Analysis of intermediates during activation into gastricsin and determination of the amino acid sequence of the propart. *Eur. J. Biochem.*, **128**, 63–70.

HENRY, J.A., MCCARTHY, A.L., ANGUS, B., WESTLEY, B.R., MAY, F.E., NICHOLSON, S., CAIRNS, J., HARRIS, A.L. & HORNE, C.H.W. (1990). Prognostic significance of the estrogen-regulated protein cathepsin D in breast cancer. *Cancer*, **65**, 265–271.

HSU, S.M., RAINE, M.L. & FANGER, H. (1981). Use of the avidin-biotin-peroxidase complex (ABC) in immunoperoxidase techniques. *J. Histochem. Cytochem.*, **29**, 577–580.

ISOLA, J., WEITZ, S., VISAKORPI, T., HOLLI, K., SHEA, N., KHABBAZ, N. & KALLIONIEMI, O.P. (1993). Cathepsin D expression detected by immunohistochemistry has independent prognostic value in axillary node-negative breast cancer. *J. Clin. Oncol.*, **11**, 36–43.

LIOTTA, L.A. (1988). Gene products which play a role in cancer invasion and metastasis. *Breast Cancer Res. Treat.*, **11**, 113–124.

MCCARTY, K.S. Jr, SZABO, E., FLOWERS, J.L., COX, E.B., LEIGHT, G.S., MILLER, L., KONRATH, J., SOPER, J.T., BUDWIT, D.A., CREASMAN, W.T., SEIGLER, H.F. & MCCARTY, K.S. SR. (1986). Use of a monoclonal anti-estrogen receptor antibody in the immunohistochemical evaluation of human tumors. *Cancer Res.*, **46**, 4244s–4248s.

MONTEAGUDO, C., MERINO, M.J., SAN-JUAN, J., LIOTTA, L.A. & STETLER-STEVENSON, W.G. (1990). Immunohistochemical distribution of type IV collagenase in normal, benign, and malignant breast tissue. *Am. J. Pathol.*, **136**, 585–592.

ROCHEFORT, H., CAPONY, F., GARCIA, M., CAVAILLES, V., FREISS, G., CHAMBON, M., MORISSET, M. & VIGNON, F. (1987). Estrogen-induced lysosomal proteases secreted by breast cancer cells. A role in carcinogenesis?. *J. Cell Biochem.*, **35**, 17–29.

SAMLOFF, I.M. (1989). Peptic ulcer: the many proteinases of aggression. *Gastroenterology*, **96**, 586–595.

SANCHEZ, L.M., FREIJE, J.P., MERINO, A.M., VIZOSO, F., FOLTMANN, B. & LOPEZ-OTIN, C. (1992*a*). Isolation and characterization of a pepsin C zymogen produced by human breast tissues. *J. Biol. Chem.*, **267**, 24725–24731.

SANCHEZ, L.M., VIZOSO, F., DIEZ-ITZA, I. & LOPEZ-OTIN, C. (1992*b*). Identification of the major protein components in breast secretions from women with benign and malignant breast diseases. *Cancer Res.*, **52**, 95–100.

SANCHEZ, L.M., FERRANDO, A.A., DIEZ-ITZA, I., VIZOSO, F., RUIBAL, A. & LOPEZ-OTIN, C. (1993). Cathepsin D in breast secretions from women with breast cancer. *Br. J. Cancer*, **67**, 1076–1081.

SAPPINO, A.P., BUSSO, N., BELIN, D., VASSALLI, J.D. (1987). Increase of urokinase type plasminogen activator gene expression in human lung and breast carcinomas. *Cancer Res.*, **47**, 4043–4046.

SLOANE, B.F., DUNN, J.R. & HONN, K.V. (1981). Lysosomal cathepsin B: correlation with metastatic potential. *Science*, **212**, 1151–1153.

SPYRATOS, F., MAUDELONDE, T., BROUILLET, J.P., BRUNET, M., DEFRENNE, A., ANDRIEU, C., HACENE, K., DESPLACES, A., ROUESSE, J. & ROCHEFORT, H. (1989). Cathepsin D: an independent prognostic factor for metastasis of breast cancer. *Lancet*, **8672**, 1115–1118.

TANDON, A.K., CLARK, G.M., CHAMNESS, G.C., CHIRGWIN, J.M. & MCGUIRE, W.L. (1990). Cathepsin-D and prognosis in breast cancer. *N. Engl. J. Med.*, **322**, 297–302.

TOLIVIA, J. & TOLIVIA, D. (1985). A new technique for differential and simultaneous staining of nerve cells and fibers. *J. Neurosc. Methods*, **13**, 305–311.

VAITUKAITIS, J.L. (1981). Production of antisera with small doses of immunogen: multiple intradermal injections. *Methods Enzymol.*, **73**, 46–52.

WINSTANLEY, J.H.R., LEINSTER, S.J., COOKE, T.G., WESTLEY, B.R., PLATT-HIGGINS, A.M. & RUDLAND, P.S. (1993). Prognostic significance of cathepsin-D in patients with breast cancer. *Br. J. Cancer*, **67**, 767–772.